# Histomorphology and Immunohistochemistry of a Congenital Nephromegaly Demonstrate Concurrent Features of Heritable and Acquired Cystic Nephropathies in a Girgentana Goat (*Capra falconeri*)

**DOI:** 10.1155/2021/8749158

**Published:** 2021-01-19

**Authors:** Christian Mayer, Steffen Ormanns, Monir Majzoub-Altweck

**Affiliations:** ^1^Center of Clinical Veterinary Medicine, Institute for Veterinary Pathology, Ludwig-Maximilians-University, Veterinärstraße 13, Munich 80539, Germany; ^2^Institute of Pathology, Ludwig-Maximilians-University, Thalkirchner Straße 36, Munich 80337, Germany

## Abstract

Polycystic kidney diseases (PKD) represent frequent congenital and adult nephropathies in humans and domestic animals. This report illustrates an uncommon state of congenital PKD in a girgentana goat (*Capra falconeri*). A stillborn female goat kid was submitted for postmortem examination and underwent macroscopic and microscopic examination. The kidneys showed a bilateral nephromegaly and a perpendicular polycystic altered texture of the renal parenchyma. Renal tissue sections were comprehensively investigated by histopathology (overview and special stains), immunohistochemistry (CD10, CD117, pan-cytokeratin, cytokeratin 7, E-cadherin, Pax2, Pax8, and vimentin), and electron microscopy (SEM, TEM). Histopathology of renal tissue sections revealed polycystic alterations of the renal parenchyma as well as conspicuous polypoid proliferates/projections of the renal tubular epithelium, which showed clear cell characteristics. Furthermore, epithelial projections were indicative for epithelio-mesenchymal-transition, cellular depolarization, and strong expression of differentiation markers Pax2, Pax8, and CD10. Ultrastructural morphology of the projections was characterized by numerous diffusely distributed, demarked round cytoplasmic structures and several apico-lateral differentiations. Additionally, hepatic malformations comprising biliary duct proliferation with saccular dilation and bridging fibrosis were observed. Notably, this report describes the first case of a congenital cystic nephropathy with overlapping features of heritable and acquired nephropathies in any species. Epithelio-mesenchymal-transition and altered cadherin expression seem to be crucial components of a suspected pathomechanism during cystogenesis.

## 1. Introduction

Polycystic kidney diseases (PKD) represent the most common renal disorder in domestic animals and humans comprising hereditary, syndromic, and acquired renal disorders and dysplasia, respectively. The heritable forms of human PKD are subdivided into a more frequently (78%) occurring autosomal dominant PKD (ADPKD), hosting a mutation of the PKD1/PKD2 gene (polycystin-1 and 2) and usually manifest in later adulthood, featuring a slowly progressive form of PKD. Furthermore, ADPKD also occurs in Persian cats (*Felis catus*), hosting a mutation in the feline PKD-1 gene [1]. The infrequent (13%) human autosomal recessive PKD (ARPKD) is characterized by a mutation of the PKHD1 gene (fibrocystin). In contrast, ARPKD features a juvenile/congenital disorder, resulting in a highly progressive severe nephromegaly, and causes abortions or early infantile death due to renal insufficiency [2]. ARPKD has been described in several animal species such as Persian kittens (*Felis catus*), sheep lambs (*Ovis aries*), and Cairn Terrier and West Highland White Terrier puppies (*Canis famliliaris*) [3–6]. A causative mutation for ARPKD in domestic animals has not been detected yet. A specific genetic background/origin remains unclear in the majority of congenital cystic nephropathies.

During the last decades, intense investigations concerning the molecular pathogenesis of PKD revealed polycystin to be a crucial regulator of cation channels within the renal tubular epithelium, whereas fibrocystin represents an integral membrane protein. Both are associated with the primary cilia of the tubular epithelium, which consists of an extracellular axoneme as well as an intracellular centrosome and operates as a mechanosensor for supraepithelial physical attractions (e.g., composition and quantity of fluid flow). The primary cilia subsequently orchestrate cell proliferation, polarization, and differentiation of the renal tubular epithelial cells via calcium-dependent second messenger signaling pathways, including mitogen-activated protein kinases or Ras [7]. A dysfunction of primary cilia proteins and signaling pathways seems to be a crucial factor for cystogenesis via aberrant secretion activities, increased cell proliferation, and altered cell polarities. These disorders thereby are termed as ciliopathies [7–9]. However, apart from its renal expression pattern, cystins are also expressed in the biliary tract epithelium. Consequently, ARPKD can emerge with cystic diseases of other organs such as liver or pancreas. This particular pathology results in an embryonal malformation of the ductal plate at different levels of the biliary tree and is termed as hepatorenal fibrocystic disease [10]. Congenital polycystic kidney disease and/or hepatorenal fibrocystic disease were observed in several wildlife ruminant species including roa deer and springbok and in several caprine species such as pygmy goat (*Capra aegagrus hircus*) and nubian goat (*Capra hircus*) [11–14].

In addition to heritable influences, cystic kidney diseases can also be acquired (acquired cystic kidney disease (ACKD)). In humans, ACKD and end-stage kidneys frequently are associated with long-term hemodialysis. Occasionally, renal cell carcinomas (RCC) or tubulocystic renal cell carcinomas occur in patients with ACKD. They presumably arise of atypical renal cysts, which are characterized by double-layered cyst epithelium and several epithelial papillary projections [15, 16].

However, little is known about emergence and pathogenesis of heritable and acquired cystic nephropathies in domestic animals and wildlife species. Here, we describe a comprehensive morphological (histopathological, immunohistochemical, and ultrastructural) investigation of this uncommon congenital disorder in the girgentana goat (*Capra falconeri*).

## 2. Case Presentation

### 2.1. Animals and Tissue Sampling

Two stillborn girgentana goat kids from a German wildlife park were submitted to the Institute for Veterinary Pathology for postmortem examination. The contributors mentioned no anomalies regarding course of gestation, birth, and pre- and perinatal constitution of the ewe. Both animals underwent comprehensive macroscopic and histologic examination. Numerous tissue samples of both animals were taken according to standard protocols and processed as indicated below.

### 2.2. Processing for Histopathological, Immunohistochemical, and Ultrastructural Examination

For histopathological examination, sections of formalin (7% formaldehyde) fixed and paraffin-embedded (FFPE) tissue samples were routinely processed and stained with hematoxylin and eosin (HE), Masson's Trichrome (MT), and periodic acid Schiff-reaction (PAS) according to standard protocols. For immunohistochemical examination, sections of FFPE-material were routinely processed and incubated with the following antibodies according to the manufactures instructions: anti-CD10 (SP-67, Roche, Basel, Switzerland), anti-CD117/c-Kit (A4502, Agilent Dako, Santa Cruz, USA), anti-pan-cytokeratin (M3515, Agilent Dako), anti-cytokeratin 7 (OV-TL12/30, Cellmarque, Rockling, CA, USA), anti-E-cadherin (610181, BD Biosciences, San Jose, CA, USA), anti-Pax2 (EPR-8586, Abcam, Cambrige, United Kingdom), anti-Pax8 (MRQ-50, Cellmarque), anti-vimentin (M0725, Agilent Dako), rabbit anti-mouse IgG/HRP (P0161, Agilent Dako), and goat anti-rabbit IgG/HRP (P0447, Agilent Dako). Standardized FFPE sections were used for positive controls. Negative controls were incubated with dilution buffer instead of primary antibodies. For visualization, the Envision mouse/rabbit (Agilent Dako Envision) and Delafield's hematoxylin counterstain were used. Pictures were taken using an orthoplan light microscope (851465, Leica Biosystems, Wetzlar, Germany) and the pathoZoom software (Smart in Media GmbH, Köln, Germany). For SEM, samples of kidney tissue were fixed in 6.25% glutaraldehyde, dehydrated, critical point dried, and sputter-coated with gold. Investigation was performed using a digital scanning electron microscope (Zeiss DSM 950, Jena, Germany). Epon embedded tissue samples were prepared for ultrathin sections, stained according to standard procedures, and underwent TEM investigation by using a transmission electron microscope (Zeiss EM 10, Jena, Germany).

## 3. Results

### 3.1. Gross Pathology

**Figure 1:**
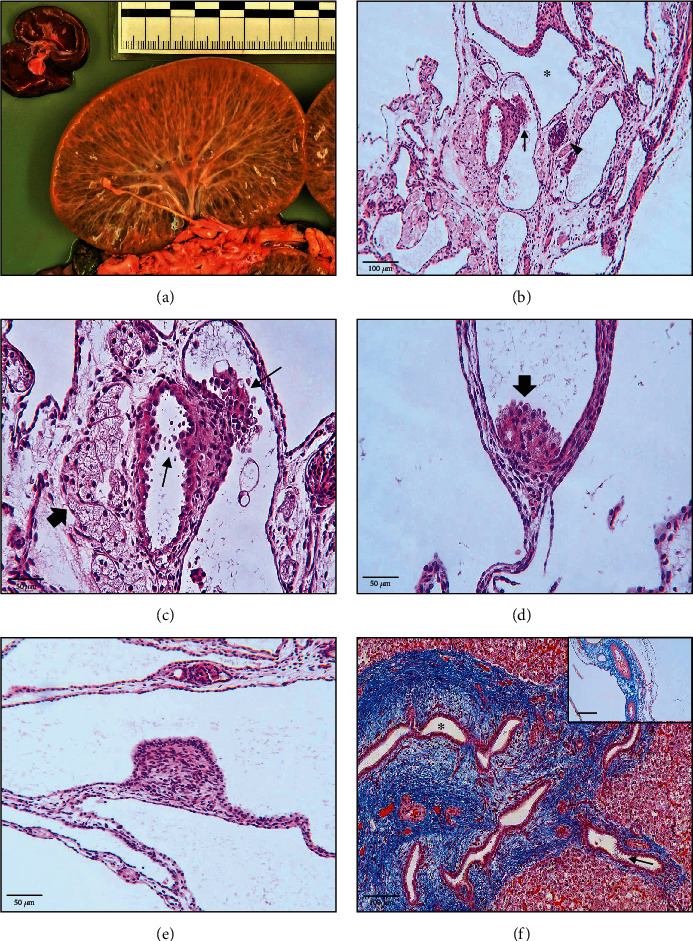
Macroscopic and microscopic features of renal and hepatic tissue. Kidney (a–e): (a) severe diffuse perpendicular polycystic alteration of the renal parenchyma. The discrimination of cortex and medulla fails. Normal sized twin kidney for scale (upper left corner). (b, c) Polycystic alteration of the renal parenchyma (asterisk) is lined by a flattened epithelium. Multifocal polypoid proliferates/projections of the tubular epithelium can be seen (thin arrows). Furthermore, there are single morphologically matured glomeruli (arrowhead). The interstitial tissue is characterized by a delicate fibrovascular stroma, blood vessels, and numerous atrophic/necrotic tubules (thick arrow). Hematoxylin and eosin (HE) (d) polypoid proliferates/projections (arrow) consist of protruding round to ovoid cells, occasionally occurring with a foamy/vacuolated pale eosinophilic cytoplasm. HE (e) fibrous protrusion lined by isoprismatic epithelium is obvious. HE. Liver: (f) multifocal proliferated and dilated biliary ducts (asterisk), surrounded by marked pericholangiolar bridging fibrosis (green). Occasionally, supraepithelial granular structures can be seen (arrow). Numerous blood vessels occur with a marked perivascular fibrosis (inset). Masson's Trichrome.

Gross examination of the goat kids revealed a fetal crown-rump length of 39 cm and 38 cm (approximately 150 days of gestation were calculated using Keller's formula), respectively. The female kid attracted with bilateral pale and enlarged kidneys (nephromegaly, maximum dimensions of 10 × 7 × 5cm). The surface occurred irregularly bumpy, smooth, and slightly fluctuating. The renal parenchyma was entirely replaced by numerous variably sized thin-walled cysts filled with clear yellow fluid (Figure 1(a)). Furthermore, the liver tissue showed multifocal to coalescing white solidified areas. Apart from autolytic changes, the male twin lamb showed no pathologic gross anomalies.

### 3.2. Histopathology

Large sized cysts, lined by a flattened, rarely cuboidal to prismatic epithelium, replaced the entire renal parenchyma (Figure 1(b)). Corresponding to the macroscopic findings a subdivision in medullar and cortical compartments failed. The cysts contained small amounts of a flocking, amorph, nonchromatic material. There were few morphologically normal, matured glomeruli with visible, rarely indistinct capillary loops. Occasionally large intraluminal, polypoid proliferates/projections of the tubular epithelium were observed, consisting of round to ovoid cells with a central round, hyperchromatic nucleus, indistinct cell borders, and a pale eosinophilic, slightly foamy/vacuolated cytoplasm (Figures 1(c) and 1(d), C&D). Several areas showed conspicuous supraepithelial granular, extracellular material (Figures 1(b) and 1(f), arrows). Furthermore, several protrusions of the cyst wall into the cyst lumina could be observed, which were characterized by an isoprismatic epithelium covering a fibrovascular stroma (Figure 1(e)). The interstitial tissue consisted of numerous atrophic/necrotic tubules, blood vessels, and several fibrocytes (Figures 1(b)–1(d)). Masson's trichrome showed delicate fibrillary collagenous fibers within the interstitial tissue as well as several blood vessels, which were affected by moderate to marked perivascular fibrosis (Figure 1(f), inset). Liver: almost 20% of the hepatic parenchyma was affected by marked hyperplasia and extensive saccular dilation of biliary duct clusters, which were surrounded by multifocal to coalescing, increased amounts of fibrous connective tissue (Masson's Trichrome, Figure 1(f)). Corresponding to the findings within renal cyst walls, supraepithelial granular, extracellular material was also seen within biliary ducts. Additionally, hepatic parenchyma was characterized by diffuse hyperemia and multifocal extramedullary hematopoiesis. Hepatocytes presented with a pale eosinophilic, foamy cytoplasm indicating glycogen storage (PAS-reaction positive).

### 3.3. Immunohistochemistry

**Figure 2:**
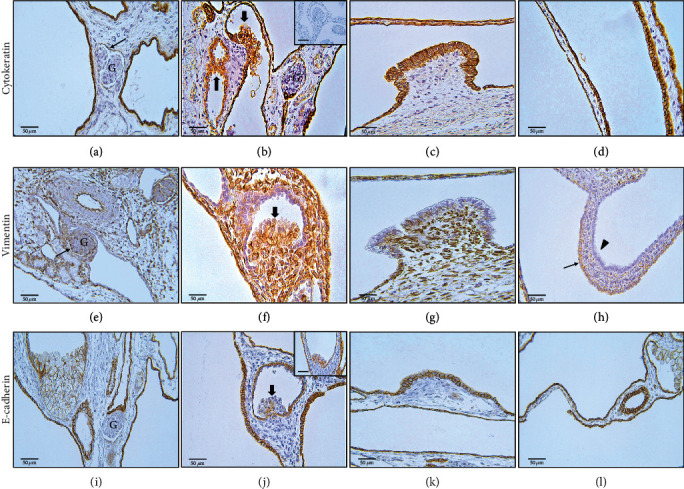
Expression of vimentin, cytokeratin, and E-cadherin (immunohistochemistry). Cytokeratin (a–d): (a) no visible cytokeratin expression within glomerular structures (G), single pericytes weakly express cytokeratin (arrow). (b) Homogenous expression pattern within the polypoid proliferates/projections, which additionally lack expression of cytokeratin 7 (inset). (c, d) Protrusions and the epithelial lining of the cysts feature a homogenous expression pattern. Vimentin (e–h): (e) vimentin gets expressed in the podocytes (arrow) of the glomeruli (G). (f) Furthermore, it is homogenously expressed in the polypoid proliferates and indicates a divergent expression pattern in protrusions (g) and cyst wall linings (h), showing positive (arrow) and negative sections (arrowhead). E-cadherin (i–l): (i) there is no E-cadherin expression within glomerular structures (G). (j) The epithelial proliferates/projections occur with a complete or apical (inset) loss of E-cadherin, sometimes associated with a basal displacement of E-cadherin expression. Cell morphology is characterized by a marked foamy cytoplasmic texture. E-cadherin mainly is expressed in intra- and intercellular areas of the protrusions (k) and the cyst wall lining (l).

**Figure 3:**
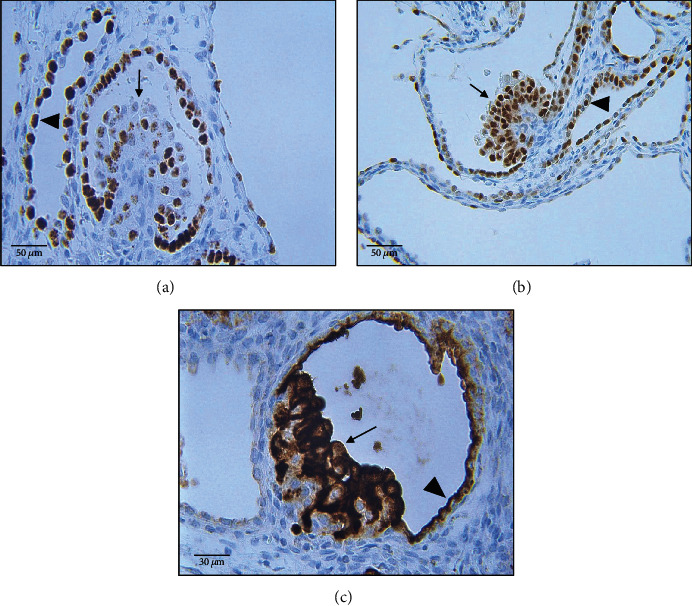
Expression of differentiation markers Pax2, Pax8, und CD10 (immunohistochemistry). PAX2 (a) homogenous expression in the tubular epithelium (arrowhead) and to lesser extent in the epithelial proliferates/projections (arrow). PAX8 (b) expression features a homogenous pattern within tubular epithelium (arrowhead) and epithelial proliferates/projections (arrow). CD10 (c): strong expression of CD10 within epithelial proliferates (arrow) and weak expression in surrounding epithelial linings (arrowhead).

Staining for cytokeratin showed a strong homogenous expression pattern in the epithelial linings of the cyst walls, the protrusions, and in the polypoid projections of the cyst wall epithelium. Cytokeratin 7 was neither expressed within proliferates nor tubular epithelium. Several pericytes weakly expressed cytokeratin (Figures 2(a)–2(d)). Vimentin staining revealed inconsistent signals in the epithelial cyst linings and homogenous signals in the polypoid projections of the tubular epithelium. Epithelial linings of the protrusions were negative for vimentin. Furthermore, several podocytes expressed vimentin (Figures 2(e)–2(h)). E-cadherin staining presented with a homogenous, predominantly intercellular expression pattern in the epithelial cyst lining and the protrusions, whereas the polypoid projections partially showed a complete loss of E-cadherin expression, particularly in the apical areas. Occasionally, the basal areas of the projections showed a weak expression of E-cadherin. E-cadherin was not expressed in cellular structures of the glomeruli (Figures 2(i)–2(l)). Most renal epithelial cells as well as the projections express the differentiation markers paired-box-protein Pax2 and Pax8 (Figures 3(a) and 3(b)). Cluster of differentiation 10 (CD10, Neprilysin) was strongly expressed in proliferates and to a lesser extent in the tubular epithelium (Figure 3(c)). They did not express CD-117/c-Kit (data not shown).

### 3.4. Ultrastructural Findings (Breakage Preparation, SEM, and TEM)

**Figure 4:**
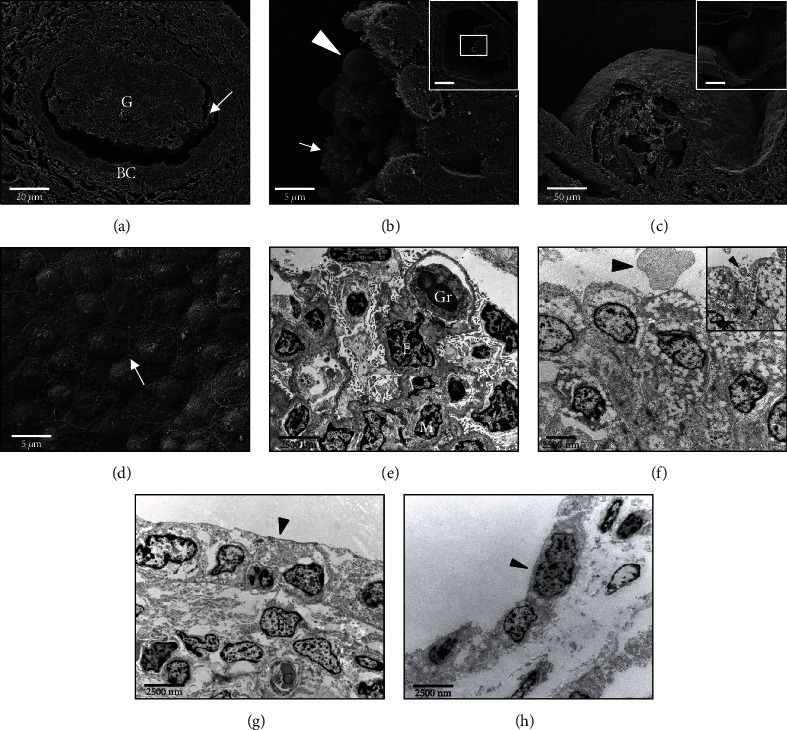
Ultrastructural findings of the renal tissue (SEM, TEM). Nephrons (a, e): (a) matured nephrons show normal structured glomerulum with capillary loops (arrow) and Bowman's capsule (BC). (e) Correspondingly, TEM reveals granulocytes (Gr) within glomerular capillaries, surrounded by podocytes (P), mensangial cells (M), and endothelial cells (E). Epithelial proliferates/projections (b, f): (b) proliferates show several apical differentiations comprising microplicae/microfilaments (arrow) and supracellular granular constrictions (arrowhead). (f) Microplicae and granules are also obvious in TEM sections (arrowheads). Cell somata show numerous diffusely distributed round, demarked cytoplasmic structures and few organelles. Intercellular areas are characterized by several desmosomes (inset, arrows). Protrusions (c, g): (c) SEM shows a cobblestone-like, slightly convex cellular relief. (g) Epithelial surfaces of the protrusions show a predominantly cuboidal epithelium without visible apical structures (arrowhead). The underlying fibrovascular tissue is characterized by several fibroblasts (F) and capillaries (C). Cyst (d, h): (d) cyst wall epithelium appears similar to epithelial linings of the protrusions and mainly consists of a flattened and slightly convex epithelium. There are numerous intercellular formations of microplicae (arrow). (h) The epithelium predominantly appears flat and without any visible apical structures (arrowhead).

SEM and TEM showed several matured glomeruli with capillaries and associated endothelial cells, intravasal blood cells (erythrocytes, granulocytes), and podocytes and mesangium cells (Figures 4(a) and 4(e)). The polypoid projections showed supracellular, granular up to round structures and several apical differentiations including microplicae/microfilamentous formations (Figure 4(b)). The cell somata had a round to ovoid nucleus with a finely granulated karyoplasm and few, homogenously distributed heterochromatin (karyosomal). There were numerous delimited, round, cytoplasmic structures and few organelles, predominantly mitochondria and endoplasmic reticulum (Figure 4(f)). The cell borders hold numerous desmosomes (Figure 4(f), inset). The epithelial linings of protrusions and cyst wall are characterized by a cuboidal to flat morphology and possessed no visible apical structures (Figures 4(c), 4(d), and 4(g)). Exclusively in intercellular areas of the cyst wall several microplicae could be observed (Figure 4(h)).

## 4. Discussion

To the best of our knowledge, this is the first report of a case of congenital cystic nephropathy with overlapping features of heritable and ACKD in any species. Bilateral nephromegaly, perpendicular cystic alteration of the renal parenchyma (collecting duct system) and hepatic fibrosis are characteristic features of heritable PKD in humans and animals, whereas epithelial proliferates/projections within the cysts are characteristics of ACKD [8, 17].

Immunohistochemical features confirm the hypothesis that compromised cell polarity and epithelio-mesenchymal-transition (EMT) are key features of pathogenesis and cystogenesis in cystic nephropathies, respectively [18, 19]. The cell polarization comprises its spatial orientation and has crucial influences on functionality, directed secretion activity (Na/K-ATPases), or expression of apical structures (microvilli). The loss of the apico-basal cell polarization is required in the process of EMT, which is characterized by a temporarily altered expression pattern of mesenchymal (vimentin) and epithelial (cytokeratin) marker proteins as well as the downregulation of cytoskeletal components and adhesion proteins such as E-cadherin. EMT and altered cadherin expression occur in progression of tumors and differentiation of embryonic cells (neural crest) and can be induced by incubation of epithelial cells with *Echinococcus granulosus* cyst fluid resulting in cyst formation [20, 21]. Furthermore, E-cadherin expression correlates with the amount and distribution of Na/K-ATPases within epithelial cells and possibly plays a role in pathogenesis and cystogenesis via aberrant secretion activity [22–24]. The cadherin-dependent baso-lateral to apical translocation of the Na-K-ATPases is a crucial mechanism for cyst formation in rodent PKD models [25, 26]. Even more, in vivo and in vitro studies revealed a reduction of cyst size in murine ADPKD by downregulation of microRNAs-192 and -194, which are responsible for EMT regulation via E-cadherin expression [27]. In the present case, positive staining for cytokeratin and formation of desmosomes confirm the epithelial origin of proliferates; therefore, vimentin expression within epithelial cells may be indicative for EMT. E-cadherin loss within proliferates may be associated with the mechanisms described above. On one hand, Pax2, Pax8, and CD10 expression indicates a renal epithelial origin of proliferates. On the other hand, strong expression of these transcription factors is also associated with migration, proliferation, stem cell maintenance, and tumor growth [28, 29]. Apical cell differentiations may show epithelial metaplasia including cystadenomatous, oncocytic, ciliated, or apocrine features as observed in acquired cystic diseases such as intraoral salivary glands in humans [30]. The conspicuous round cytoplasmic structures may show lipid droplet storage or other cytoplasmic/paraplasmic inclusions (secretory granules, glycogen).

Proliferates/papillary projections of the renal tubular epithelium are frequently observed in human medicine and are associated with ACKD, especially in patients receiving long-term hemodialysis [16]. These lesions are supposed to be precancerous epithelial changes in end-stage kidneys and can lead to RCC, mostly clear cell carcinomas [31]. However, cytomorphology (pale, slightly vacuolated cytoplasm, ultrastructural cytoplasmic inclusions, and few organelles) and immunohistochemical pattern (expression of cytokeratin, vimentin, Pax2, Pax8, and CD10 and a lack of cytokeratin 7, CD117, and E-cadherin expression) of the projections resemble clear cell characteristics [32].

Differentials for cystic nephropathies include ADPKD, multicystic and glomerulocystic diseases, and obstructive lesions. ADPKD is unlikely due to the almost exclusively restricted incidence in adult individuals, and characteristic histologic features include marked interstitial fibrosis with lymphoplasmacytic infiltration. Multicystic diseases are characterized by the loss of renal morphology and severe developmental changes including undifferentiated mesenchyme and metaplastic cartilage. Glomerulocystic diseases occur in syndromic PKD, comprising von Hippel-Lindau syndrome and tuberous-sclerosis-complex, which are marked by renal and extrarenal neoplasms. Furthermore, no obstructions of the urinary tract were found, which probably rules out obstruction-associated PKD [33].

Pulmonary hypoplasia due to renal oligohydramnios represents a major reason for neonatal complications, high mortality, and long-term morbidity of ARPKD in humans and domestic animals [34]. Interestingly, this symptom was not observed in this case. We postulate compensation mechanisms of the twin due to the structure of the ruminant synepitheliochorial placentation and the early formation of placental anastomoses [35, 36].

Several animal models, including murine and ovine species have been established for specific research issues of PKD in humans [37]. Ruminant models provide several advantages in terms of genetics, metabolism, surgical procedures, and pharmacologic treatments of perinatal diseases [38]. TMEM67-gene was figured out to host the causative mutation of Meckel's syndrome in humans using an ovine research model [39]. These studies emphasize the relevance of comprehensive and comparative investigations of PKD. In summary, we demonstrated several indications that EMT and cellular depolarization are crucial in pathogenesis of congenital and/or acquired PKD in goats [40]. The morphologic and ultrastructural findings mostly resemble neonatal/juvenile PKD (sensu strictu) with overlapping features of acquired cystic kidney diseases and clear cell characteristics. This state has not been described in any further species yet. The relevance in terms of pathogenesis, genetics, and specific cell morphology needs to be further elucidated.

## Data Availability

Readers can get access to supporting data by request and contacting the corresponding author (Dr. Christian Mayer) or the academic director of the Institute for Veterinary Pathology (Dr. Monir Majzoub-Altweck) using the indicated mailing addresses.
